# Effects of Short‐Term Intensive Insulin Therapy Combined With Oral Hypoglycemic Agents for Inducing Remission in Newly Diagnosed Type 2 Diabetes Mellitus: A Randomized Clinical Trial

**DOI:** 10.1111/1753-0407.70187

**Published:** 2026-01-11

**Authors:** Weijian Ke, Liehua Liu, Pengyuan Zhang, Li Yan, Qian Zhang, Fan Zhang, Xuejuan Xu, Juan Liu, Lijuan Xu, Xuesi Wan, Hai Li, Xiaopei Cao, Haipeng Xiao, Melissa S. Putman, Yanbing Li

**Affiliations:** ^1^ Department of Endocrinology The First Affiliated Hospital, Sun Yat‐sen University Guangzhou Guangdong Province China; ^2^ Department of Endocrinology and Metabolism Sun Yat‐sen Memorial Hospital, Sun Yat‐sen University Guangzhou Guangdong Province China; ^3^ Department of Endocrinology Southern Medical University Nanfang Hospital Guangzhou Guangdong Province China; ^4^ Department of Endocrinology Peking University Shenzhen Hospital Shenzhen Guangdong Province China; ^5^ Department of Endocrinology The First People Hospital of Foshan Foshan Guangdong Province China; ^6^ Diabetes Research Center Massachusetts General Hospital Boston Massachusetts USA

**Keywords:** continuous subcutaneous insulin infusion, metformin, pioglitazone, short‐term intensive insulin therapy, sitagliptin, type 2 diabetes mellitus

## Abstract

**Aims:**

To investigate the effects of combining oral hypoglycemic agents with short‐term intensive insulin therapy (SIIT) via continuous subcutaneous insulin infusion (CSII) on glycemic outcomes in adults with newly diagnosed type 2 diabetes mellitus.

**Materials and Methods:**

In this multicenter, open‐label trial, 245 participants were randomized to three treatment arms: SIIT for 2 weeks (CSII group), SIIT plus 90‐day metformin and pioglitazone (CSII + Met + Pio group), or SIIT plus 90‐day sitagliptin (CSII + Sita group). The primary outcome was the 12‐month diabetes remission rate. Parameters of glycemic control, β‐cell function, and insulin resistance were compared among groups.

**Results:**

The participants had a mean HbA1c of 10.6% ± 2.2%. Compared to the CSII group, both combination groups had lower total daily and pre‐meal bolus insulin requirements with higher time in tight target range (TITR) during SIIT, and had significantly greater acute insulin response (AIR) after SIIT. Three months post SIIT, more participants in the CSII + Met + Pio group (78.7%, 59/75) achieved HbA1c < 6.5% compared with the CSII group (59.0%, 46/78; adjusted *p* < 0.05), but the 12‐month diabetes remission rates were similar (*p* = 0.972).

**Conclusions:**

Oral hypoglycemic agents facilitated SIIT implementation and enhanced transient improvements in glycemic control. However, similar 12‐month diabetes remission rates suggest prolonged sequential therapy may be needed for sustained glycemic benefit.

## Highlights

A 3‐month regimen of insulin sensitizers (metformin and pioglitazone) or sitagliptin, combined with short‐term intensive insulin therapy (SIIT), promoted tight glycemic control during SIIT period and improved short‐term glycemic parameters and β‐cell function. However, these benefits did not translate into enhanced long‐term diabetes remission. Further studies are warranted to evaluate whether extended sequential therapies targeting insulin resistance and obesity can achieve superior long‐term glycemic control and β‐cell preservation.

## Graphical abstract

The addition of insulin sensitizers (metformin and pioglitazone) or sitagliptin to short‐term intensive insulin therapy (SIIT) facilitated SIIT implementation and enhanced short‐term glycemic control in patients with newly diagnosed type 2 diabetes. However, 12‐month remission rates were similar to SIIT alone, suggesting a need for prolonged therapy.

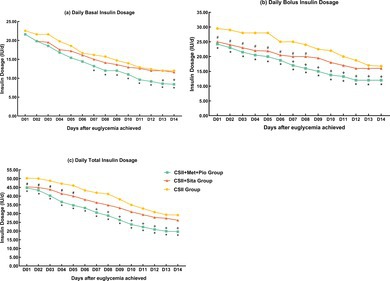

## 1 Introduction

Type 2 diabetes mellitus is characterized by progressive hyperglycemia resulting from the dysfunction of pancreatic β cells and insulin resistance. In China, diabetes imposes a substantial and rising burden, with adverse effects on quality of life and considerable economic consequences [1]. Despite the expanding therapeutic medical options, more than 50% of individuals with diabetes have suboptimal glycemic control [2], This challenge is particularly urgent in China, where adults with newly diagnosed type 2 diabetes mellitus under 65 years had mean HbA1c of 8.5% ± 2.5%, and fasting plasma glucose (FPG) is 9.5 ± 3.9 mmol/L at diagnosis [3], demonstrating the urgent need for optimizing early glucose‐lowering strategies for severe hyperglycemia. Current consensus from the American Diabetes Association (ADA) and the European Association for the Study of Diabetes (EASD) recommends combination therapy for patients with HbA1c levels exceeding 8.5%, and insulin based therapy when HbA1c levels above 10% [4]. However, standardized treatment pathways for newly diagnosed type 2 diabetes mellitus with severe hyperglycemia remains unclear [5]. Conventional therapy which stepwisely escalates glucose‐lowering agents as hyperglycemia progresses is insufficient to achieve rapid glycemic control and fails to halt β‐cell deterioration.

A growing body of evidence has shown that in early type 2 diabetes mellitus presenting with severe hyperglycemia, reversing β‐cell dysfunction could be achieved by approaches aggressively eliminating glucotoxicity. Short‐term intensive insulin therapy (SIIT) induces β‐cell recovery and drug‐free glycemic remission by maximally ameliorating glucotoxicity [6]. In our previous studies, over 50% of adults with newly diagnosed type 2 diabetes mellitus (mean HbA1c 9.7%–10%) obtained 1‐year remission after SIIT, accompanied by improvement in β‐cell function and insulin sensitivity [7, 8]. As a result, Chinese guidelines now recommend SIIT as a primary option for HbA1c > 9% at type 2 diabetes mellitus diagnosis [9].

However, remission durability remains suboptimal. The remission rate was ~60%–70% at 3‐month post SIIT, declining to 50% at 1 year and approximately 40% by year 2 [7, 8]. Notably, over 60% of the hyperglycemia relapse occurred within the first 3 months after SIIT [6]. Crucially, strict glycemic normalization is a key mechanism for β‐cell functional recovery. Studies have shown that lower mean blood glucose and higher TIR throughout the SIIT period is pivotal for reversing β‐cell dysfunction and achieving long‐term remission [10, 11]. Therefore, rapidly attaining and sustaining strict glycemic targets throughout and immediately after SIIT is crucial. This requires intensive glycemic monitoring and frequent insulin adjustments, which can impede the maximization of the SIIT therapeutic benefit. On the other hand, strategies for maintaining glycemic normalization in the period following SIIT cessation require further exploration. If a short course of oral hypoglycemic agents (OHAs) combined with SIIT could facilitate glycemic control during and after SIIT, enhanced β‐cell function and increased long‐term diabetes remission might be expected.

Insulin sensitizers and dipeptidyl‐peptidase‐4 (DPP‐4) inhibitors are first‐line OHAs. Insulin sensitizers (e.g., metformin and thiazolidinediones), reduce insulin secretion demand, thereby alleviating β‐cell metabolic stress and mitigating subsequent dysfunction or dedifferentiation [12, 13]. DPP‐4 inhibitors modulate insulin secretion in β cells and glucagon release from α cells by elevating endogenous incretins. In previous clinical studies, both insulin sensitizers and DPP‐4 inhibitors significantly improved glycemic control in insulin‐treated patients, which may be beneficial for optimizing β‐cell response to SIIT [14]. In this randomized clinical trial, we hypothesize that supplementing SIIT for 3 months with insulin‐sensitizing medications (metformin plus pioglitazone) or a DPP‐4i (sitagliptin) will facilitate SIIT implementation and improve both short‐ and long‐term glycemic outcomes versus SIIT alone.

## 2 Materials and Methods

### 2.1 Participants

Adults newly diagnosed with type 2 diabetes mellitus according to the 1999 World Health Organization diagnostic criteria [15] with no prior usage of anti‐hyperglycemic agents were recruited from five diabetes centers in China. The eligibility criteria included 25–65 years of age, BMI of 21–35 kg/m^2^, and FPG between 7.0 and 16.7 mmol/L. Participants were excluded if they were positive for autoimmune antibodies against islets, had severe acute or chronic diabetes complications, had any severe concurrent disease, or were using drugs that affect glucose homeostasis. The study protocol was approved by the Medical Research and Ethics Committee of the First Affiliated Hospital of Sun Yat‐sen University (Guangzhou, China), and all enrolled participants gave informed consent. This study was registered at ClinicalTrials.gov with registration identifier number NCT01471808.

### 2.2 Study Design

All participants were admitted to a participating hospital for SIIT consisting of continuous subcutaneous insulin infusion (CSII) for a 2‐week period. Participants were assigned to one of the following three groups (CSII alone, CSII + Met + Pio, or CSII + Sita group) by sequentially opening sealed envelopes arranged in a computer‐generated random order. Participants and investigators were not blinded to treatment assignment.

For SIIT, all participants received insulin lispro (Humalog, Eli Lilly, USA) or insulin aspart (NoveRapid, Novo Nordisk, Denmark) with an insulin pump (MiniMed 712, Medtronic, Northridge, CA). The initial insulin dosage was 0.5 IU/kg/day, with the total daily dose divided equally into basal and bolus doses. Capillary blood glucose values were measured at least seven times daily (before and 2 h after each meal, bedtime). Basal infusion rate and pre‐meal boluses were titrated to achieve euglycemia (fasting glucose < 6.1 mmol/L; postprandial glucose < 8.0 mmol/L), following the recommendations of the 2010 Chinese Guidelines for Insulin Pump Therapy [16]. In the CSII + Met + Pio group, participants received metformin (Glucophage, Bristol‐Myers Squibb/Shanghai Squibb, China) 500 mg three times per day and pioglitazone (Actos, Takeda, Japan) 30 mg once per day in addition to CSII. In the CSII + Sita group, participants received sitagliptin (Januvia, Merck Sharp & Dohme, Australia) 100 mg once daily in addition to CSII. After glycemic targets were achieved and maintained for 14 days, CSII was discontinued. Metformin, pioglitazone (the CSII + Met + Pio group), or sitagliptin (the CSII + Sita group) were continued for 90 days.

During SIIT, participants received meals based on nutritional guidelines and were encouraged to walk or jog for 30–60 min after each meal. Meals provided 50%–60% carbohydrates, 10%–15% proteins, and 20%–30% fats, with breakfast, lunch, and dinner contributing 20%, 40%, and 40% of total calories, respectively. After leaving the hospital, participants were encouraged to continue adhering to the dietary control and regular exercise.

### 2.3 Measurements and Endpoints

Disease duration was determined by either the onset of clinical symptoms or the self‐reported period of elevated blood glucose. Weight, height, blood pressure, and waist and hip circumferences were measured at baseline. Blood samples were collected for FPG, HbA1c, fasting lipid profiles, and post‐breakfast plasma glucose. Intravenous glucose tolerance test (IVGTT) using 25 g glucose (50 mL of 50% glucose) was performed to assess first‐phase insulin secretion by calculating the acute insulin response (AIR) using serum insulin levels at 0, 1, 2, 4, 6, and 10 min. AIR was defined as the incremental trapezoidal area of insulin levels during the test. Homeostasis model assessment was used to estimate insulin resistance (HOMA‐IR), β‐cell function (HOMA‐B), and the disposition index (DI) [HOMA‐IR = FPG × fasting insulin/22.5; HOMA‐B = 20 × fasting insulin/(FPG − 3.5); DI = AIR/HOMA‐IR] [17]. Time in tight range (TITR), the percentage of time that glucose levels were between 3.9 and 7.8 mmol/L, was generated using daily seven‐point capillary blood glucose values during SIIT. The corresponding percent time above range (TAR), time below range (TBR), and mean blood glucose (MBG) were also calculated. After CSII suspension, all baseline measurements were repeated at least 15 h after cessation of insulin infusion to eliminate potential interference from exogenous insulin analogs, and then again at 3 months. During each scheduled 3‐month follow‐up visit or any interim assessment, investigators systematically evaluated participants' glycemic status and reviewed their treatment records.

The primary endpoint was the difference in the 12‐month diabetes remission rate, which was defined as a HbA1c level below 6.5% without glucose‐lowering medications among the treatment groups. The secondary endpoints included inter‐group differences in insulin dosages, TITR, TAR, and TBR during SIIT, as well as in glycemic control, β‐cell function, insulin resistance indices, and weight following SIIT and at the 3‐month follow‐up. The frequency of hypoglycemia and other adverse events was recorded.

### 2.4 Statistical Analyses

The sample size was calculated based on the assumption that the 12‐month remission rate of the CSII group, the CSII + Met + Pio group, and the CSII + Sita group were 50%, 75%, and 75%, respectively. A sample size of 74 patients in each group was required with 80% power and a type I error of no more than 0.05. Eighty‐eight patients were recruited in each group with an assumed dropout rate of 20%.

All randomized participants who completed SIIT entered follow‐up and were included in the intention‐to‐treat analysis. Missing data were handled using multiple imputation. Statistical analyses were conducted with SPSS software for Windows version 23.0. Normally distributed variables were presented as mean ± SD, and compared using one‐way ANOVA with Fisher's least significant difference (LSD) test for post hoc analyses. Non‐normally distributed variables were expressed as median (interquartile range, IQR) and compared using the Kruskal–Wallis test with Bonferroni‐adjusted pairwise comparisons. Differences in proportions were analyzed with the Chi‐square test. Kaplan–Meier curves were used to assess the time‐to‐event data for diabetes remission. A two‐sided value of *p* < 0.05 was defined as statistically significant.

## 3 Results

### 3.1 Baseline Characteristics

**FIGURE 1:**
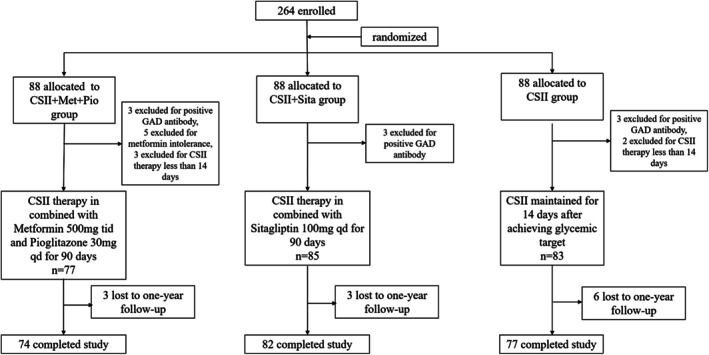
Trial profile.

Two hundred and sixty‐four patients with newly diagnosed type 2 diabetes mellitus were screened. Fourteen patients were excluded because of positive GAD antibody and metformin intolerance, and five were excluded due to not completing SIIT (Figure 1). The remaining 245 participants were included in the analyses. Baseline characteristics are described in Table 1. Mean age of participants was 47.3 ± 9.2 years, and mean HbA1c was 10.6% ± 2.2%. There were no significant differences in anthropometric data, blood glucose levels, HbA1c, lipid profiles, indices of β‐cell function (AIR, HOMA‐B, DI) and insulin resistance (HOMA‐IR) among the treatment groups at baseline (Table 1).

**TABLE 1:** Baseline characteristics.

	CSII + Met + Pio group	CSII + Sita group	CSII group	*p*
Number	77	85	83	—
Age (years), mean ± SD	46.6 ± 9.2	47.3 ± 9.2	47.5 ± 9.2	0.722
Gender (F/M)	18/59	31/54	25/58	0.193
Familial diabetes history (%)	44.2 (34/77)	50.6 (43/85)	48.2 (40/83)	0.712
Smoking (%)	37.7 (29/77)	38.8 (33/85)	37.3 (31/83)	0.979
Duration of diabetes (month)	6 (2, 12)	6 (1, 24)	6 (1, 12)	0.752
Weight (kg), mean ± SD	72.0 ± 12.2	68.6 ± 10.8	71.3 ± 11.3	0.145
BMI (kg/m^2^), mean ± SD	25.9 ± 3.0	25.3 ± 2.7	25.5 ± 2.7	0.262
Ketosis at diagnosis (%)	33.8	31.8	36.1	0.835
FPG (mmol/L), mean ± SD	11.4 ± 3.1	11.1 ± 3.2	11.6 ± 3.1	0.525
HbA1c (%), mean ± SD	10.3 ± 2.1	10.7 ± 2.3	10.7 ± 2.1	0.374
TG (mmol/L), median (IQR)	1.63 (1.24, 2.51)	1.75 (1.19, 2.73)	1.58 (1.18, 2.26)	0.569
CHOL (mmol/L), mean ± SD	5.54 ± 1.17	5.61 ± 1.13	5.39 ± 1.12	0.421
HDL‐c (mmol/L), mean ± SD	1.05 ± 0.24	1.10 ± 0.27	1.12 ± 0.25	0.273
LDL‐c (mmol/L), mean ± SD	3.70 ± 0.84	3.71 ± 0.96	3.62 ± 0.83	0.731
HOMA‐IR, median (IQR)	3.19 (2.40, 4.60)	3.30 (2.14, 4.23)	2.97 (1.78, 4.14)	0.401
HOMA‐B, median (IQR)	19.53 (9.16, 37.59)	17.78 (10.53, 37.52)	17.62 (8.32, 27.83)	0.260
AIR (μU min/mL), median (IQR)	−7.95 (−20.34, 7.28)	−5.05 (−19.13, 9.90)	−6.75 (−17.50, 1.05)	0.488
DI, median (IQR)	−2.74 (−5.82, 2.48)	−1.48 (−5.59, 2.76)	−3.41 (−5.85, 023)	0.475

Abbreviations: AIR, acute insulin response; BMI, body mass index; CHOL, cholesterol; DI, disposition index; FPG, fasting plasma glucose; HDL‐c, high‐density lipoprotein cholesterol; LDL‐c, low‐density lipoprotein cholesterol; TG, triglyceride.

### 3.2 Impact of the OHAs on Insulin Dosage and Glycemic Control During SIIT

**FIGURE 2:**
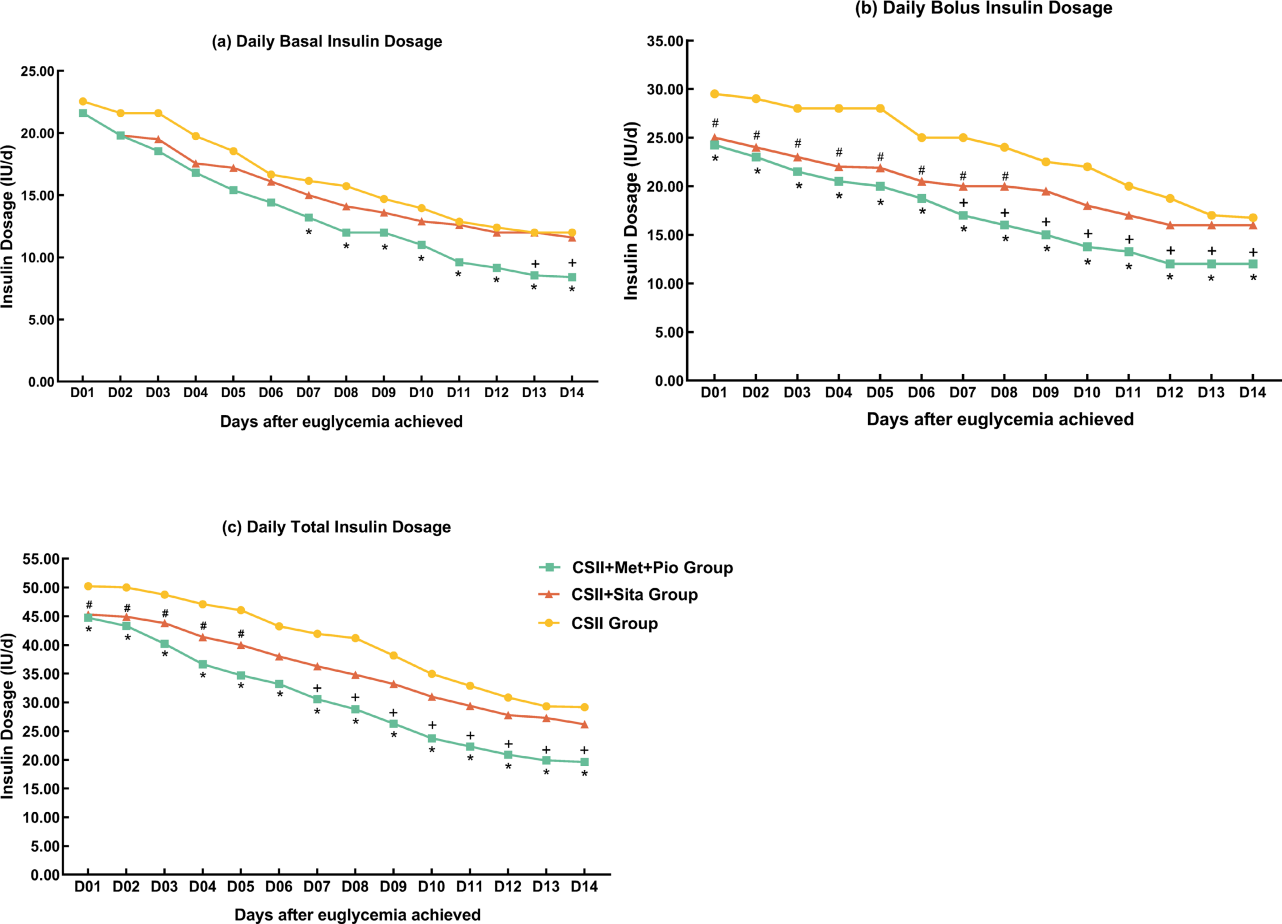
(a) Daily basal insulin dosages during SIIT. (b) Daily bolus insulin dosages during SIIT. (c) Daily total insulin dosages during SIIT, *CSII group versus CSII + Met + Pio group, *p* < 0.05, ^#^CSII group versus CSII + Sita group, *p* < 0.05, ^+^CSII + Met + Pio group versus CSII + Sita group, *p* < 0.0.05.

The time to reach glycemic targets was 2 (1, 2) days in the CSII + Met + Pio group, which was significantly shorter than that in the CSII + Sita group (3 [2, 4] days) and the CSII group (3 [1, 4] days) (*p* < 0.001 for both). On the first day upon achieving glycemic targets, both the total daily insulin dose and pre‐meal bolus insulin dose were significantly lower in the two combination groups as compared to the CSII group, while the daily basal insulin doses did not significantly differ across the three groups (*p* = 0.328) (Figure 2, Tables S1–S3). Thereafter, insulin doses declined gradually till the end of SIIT, with the CSII + Met + Pio group maintaining consistently the lowest insulin requirements, whereas doses in the CSII + Sita group gradually approached those of the CSII group (Figure 2, Tables S1–S3).

During SIIT, the combination therapy groups demonstrated superior glycemic metrics compared with CSII alone. TITR was significantly higher in both combination groups versus the CSII group (*p* < 0.001). The CSII + Met + Pio group achieved both the lowest TAR (*p* < 0.001) and MBG (*p* < 0.001) compared with the other intervention groups, while the CSII + Sita group exhibited the lowest TBR among all treatment arms (Table 2).

**TABLE 2:** Glycemic control and metabolic parameters during and after SIIT.

	CSII + Met + Pio group	CSII + Sita group	CSII group	*p*
During SIIT				
TITR (%), mean ± SD	86.87 ± 6.68	85.55 ± 6.70	79.67 ± 8.62	< 0.001
TBR (%), mean ± SD	4.03 ± 3.32	2.65 ± 2.59	4.52 ± 3.76	< 0.001
TAR (%), mean ± SD	9.10 ± 6.56	11.80 ± 6.61	15.8 ± 3.76	< 0.001
MBG (mmol/L), mean ± SD	5.8 ± 0.4	6.0 ± 0.4	6.1 ± 0.5	< 0.001
After SIIT				
FPG (mmol/L), mean ± SD	5.7 ± 0.8	6.5 ± 1.1	6.3 ± 1.0	< 0.001
HbA1c (%), mean ± SD	8.8 ± 2.0	8.9 ± 1.6	8.9 ± 1.6	0.918
AIR (μU min/mL), median (IQR)	86.60 (44.55, 130.76)	77.82 (37.62, 125.83)	53.70 (21.20, 107.74)	0.002
HOMA‐IR, median (IQR)	1.55 (1.10, 2.05)	2.10 (1.43, 2.65)	1.86 (1.31, 2.54)	0.008
HOMA‐B, median (IQR)	53.04 (35.19, 88.95)	56.11 (33.67, 71.87)	42.00 (29.80, 63.75)	0.035
DI, median (IQR)	52.70 (26.02, 96.49)	36.41 (20.18, 66.49)	24.72 (11.35, 65.51)	< 0.001
Change of weight (kg), mean ± SD	−1.2 ± 1.2	−1.0 ± 1.2	−1.2 ± 1.5	0.558

Abbreviations: AIR, acute insulin response; DI, disposition index; FPG, fasting plasma glucose; MBG, mean blood glucose; SIIT, short‐term insulin therapy; TAR, time above range; TBR, time below range; TITR, time in tight target range.

### 3.3 The Effects of Combination Therapies on Blood Glucose, β‐Cell Dysfunction, and Insulin Resistance Immediately After SIIT Completion

At SIIT completion, glycemic control was significantly improved in the overall participants, with FPG decreasing from 11.4 ± 3.2 mmol/L at baseline to 6.3 ± 1.1 mmol/L and HbA1c decreasing from 10.6% ± 2.2% to 8.9% ± 1.7% (*p* < 0.001). Additionally, β‐cell function significantly improved, with AIR increasing from −6.90 (−19.28, 5.43) to 69.00 (30.30, 120.36) μU min/mL, HOMA‐B from 17.98 (8.81, 34.11) to 50.45 (33.41, 71.34), and DI from −2.56 (−5.83, 1.90) to 38.47 (17.72, 73.68) (all *p* < 0.001). Insulin sensitivity also improved significantly, with HOMA‐IR decreasing from 3.13 (2.14, 4.28) to 1.79 (1.23, 2.49) (*p* < 0.001).

**FIGURE 3:**
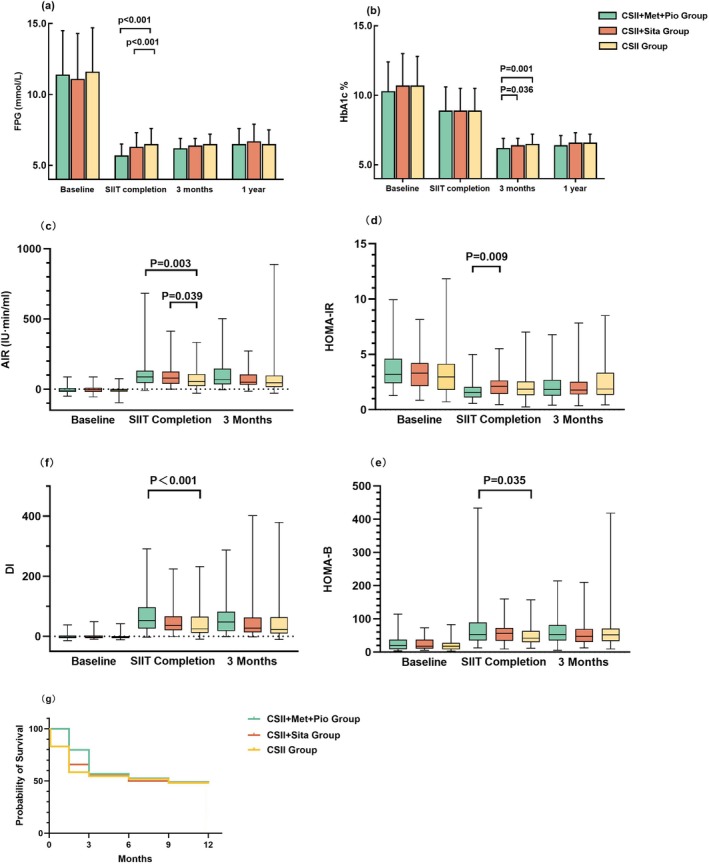
(a) FPG and (b) HbA1c levels among groups at baseline, SIIT completion, at 3‐month and 1‐year visit; among groups at baseline, CSII suspension and at 3‐month and 1‐year visit. Change of (c) acute insulin response (AIR), (d) HOMA‐IR, (e) HOMA‐B, and (f) disposition index (DI) among different treatment groups at baseline, SIIT completion and at 3‐month visit. (g) Kaplan–Meier curves of diabetes remission during follow‐up in the treatment groups.

The CSII + Met + Pio group demonstrated significantly lower post‐SIIT FPG levels compared with the other groups (both *p* < 0.001, Table 2 and Figure 3a). Additionally, HOMA‐IR was significantly lower in the CSII + Met + Pio group than in the CSII + Sita group (*p* = 0.009, Table 2 and Figure 3d). Both combination therapy groups exhibited significantly higher AIR compared to the CSII group (CSII + Met + Pio vs. CSII, *p* = 0.003; CSII + Sita vs. CSII, *p* = 0.039, Table 2 and Figure 3c). Compared to the CSII group, DI was significantly elevated (*p* < 0.001, Table 2 and Figure 3e), while HOMA‐B was significantly higher in the CSII + Met + Pio group (*p* = 0.035, Table 2 and Figure 3e). No significant differences in HbA1c levels were observed between groups at this point (Table 2 and Figure 3b).

### 3.4 Treatment Effects at the 3‐Month Follow‐Up

At the 3‐month visit when all OHAs were discontinued, HbA1c continued to decrease in all three groups, with a significantly lower level in the CSII + Met + Pio group (6.2% ± 0.7%, 6.4% ± 0.5%, and 6.5% ± 0.7%, for the CSII + Met + Pio, CSII + Sita and CSII groups, respectively, overall *p* = 0.003, Figure 3b). In the CSII + Met + Pio group, 78.7% (59/75) of participants achieved HbA1c < 6.5%, significantly higher than the CSII group (59.0%, 46/78) and the CSII + Sita group (65.9%, 54/82) (overall *p* = 0.031; adjusted *p* < 0.05 for CSII + Met + Pio group vs. CSII group and CSII + Met + Pio group vs. CSII + Sita group, respectively).

The differences in FPG, AIR, HOMA IR, HOMA‐B, and DI among the treatment groups were no longer significant (Figure 3).

### 3.5 Diabetes Remission During 12‐Month Follow‐Up

Two hundred and thirty‐three patients completed 12‐month follow‐up (74 in the CSII + Met + Pio group, 82 in the CSII + Sita group and 77 in the CSII group). As shown in Figure 3g, the primary endpoint, diabetes remission rates at 12 months, were 50.0% (37/74), 48.8% (40/82), and 50.6% (39/77) in the CSII + Met + Pio, CSII + Sita, and CSII groups, respectively, with no statistical significance between the three groups (*p* = 0.972).

### 3.6 Body Weight

All groups showed modest weight loss from baseline after SIIT with no significant differences (*p* = 0.558, Table 2). At the 3‐month follow‐up, weight reduction was observed across all treatment groups (−4.1 ± 2.4, −3.3 ± 2.9, and −2.6 ± 2.5 kg, for CSII + Met + Pio, CSII + Sita, and CSII groups, respectively; *p* = 0.002), with group CSII + Met + Pio demonstrating a significantly greater reduction compared to the control group (*p* < 0.001 vs. control). This weight loss persisted through 12‐month follow‐up with no inter‐group differences (−2.1 ± 2.9, −2.0 ± 1.7, and −2.3 ± 2.5 kg, for CSII + Met + Pio, CSII + Sita, and CSII groups, respectively; *p* = 0.738).

### 3.7 Adverse Events

The incidence of hypoglycemic episodes, defined as capillary blood glucose < 3.9 mmol/L, differed among the three treatment groups during SIIT (3 [2, 5] vs. 2 [1, 3] vs. 3 [1, 4] times for the CSII + Met + Pio, CSII + Sita, and CSII groups, respectively; *p* = 0.005), with the CSII + Sita group experiencing fewer episodes than the CSII + Met + Pio group (*p* = 0.002). All hypoglycemic episodes were corrected after ingestion of carbohydrates, and no severe hypoglycemic episodes (defined as loss of consciousness) occurred. Gastrointestinal symptoms, most of which were mild, occurred in 16.8% of patients in the CSII + Met + Pio group and 3.7% of patients in the CSII + Sita group (*p* = 0.004).

## 4 Discussion

In this randomized trial, insulin sensitizers or sitagliptin facilitated SIIT implementation by reducing insulin requirements and accelerating near‐normoglycemia achievement in adults with newly diagnosed type 2 diabetes mellitus and marked hyperglycemia. The combination therapies also improved short‐term glycemic control, β‐cell function, and insulin sensitivity compared to SIIT alone. However, benefits dissipated post‐treatment discontinuation, indicating unmodified ongoing pathophysiological processes underlying β‐cell dysfunction and hyperglycemia relapse. This necessitates novel strategies for sustained treatment effects.

Limited evidence exists regarding OHAs combination with SIIT in newly diagnosed type 2 diabetes mellitus. Our pilot study showed that 3‐month rosiglitazone or metformin improved β‐cell function and insulin sensitivity after CSII, increasing the remission rate after drug withdrawal; however, long‐term treatment response remained unknown [18]. Hence, this trial evaluated both the immediate metabolic effects and long‐term remission trajectory of combination CSII with different types of OHAs. During active treatment, combination therapies significantly improved glycemic control and β‐cell function, with CSII + Met + Pio achieving higher HbA1c < 6.5% rates. However, benefits were not sustained after treatment discontinuation, with similar 12‐month remission rates of ~50% across groups. These findings align with REMIT‐sita and REMIT‐dapa studies in early type 2 diabetes mellitus, which demonstrated that combining basal insulin with oral antihyperglycemic agents for 8–12 weeks (metformin plus DPP‐4 inhibitors or SGLT‐2 inhibitors), provided only short‐term benefits while long‐term remission rates were comparable to standard management [19, 20]. Our prior clinical study likewise demonstrated that the addition of a short‐term GLP‐1 receptor agonist (GLP‐1 RA) to IIT yielded only transient improvements in β‐cell function and glycemic control, with these effects dissipating soon after treatment discontinuation [21]. The collective evidence underscores the importance of mechanistic insights into transient versus sustained therapeutic benefits to optimize treatment strategies for achieving durable diabetes remission in early‐stage type 2 diabetes mellitus.

The major underlying mechanism of SIIT was thought to be the β‐cell restoration brought about by blood glucose normalization [7, 8, 17]. Our previous studies identified lower MBG, higher TITR during SIIT, and lower post‐SIIT FPG as predictors of long‐term diabetes remission [10, 11, 22, 23]. Animal studies demonstrate that rapid glucotoxicity relief restores insulin secretion and promotes β‐cell redifferentiation while inhibiting apoptosis [24]. Despite improvements in glycemic control and β‐cell function with OHA combination therapy, these surrogate markers did not translate into higher long‐term remission rates. This disconnect indicates that combination of OHAs provides transient metabolic benefits but does not yield sustained clinical advantage beyond that achieved with SIIT alone, suggesting that SIIT itself is sufficient to maximize β‐cell restoration in most patients with severe hyperglycemia. However, given the substantial inter‐patient variation in pathogenesis and therapeutic responses, identifying patient subtypes based on clinical characteristics that may be most likely to benefit from this intervention should be explored in the future [25]. In addition, the combination therapy facilitated SIIT implementation by reducing both time to glycemic target achievement and insulin dose requirements, with significantly improved TITR. Rigorous glycemic control during SIIT requires intensive glucose monitoring and frequent insulin adjustments, often necessitating inpatient management that may not be feasible in many healthcare systems. Thus, supplementing CSII with insulin sensitizers or DPP‐4 inhibitors might expand the application scenario of SIIT, particularly in outpatient settings. Moreover, DPP‐4 inhibitors were shown to associate with a lower incidence of hypoglycemia and gastrointestinal side effects [26], rendering them an important alternative in cases of metformin intolerance or hypoglycemia concerns.

The temporal benefits of combination intervention suggest that maintenance of treatment effects may require persistent interventions. There are multiple mechanisms contributing to hyperglycemic relapse after SIIT, including aging, ongoing metabolic dysregulation, weight regain, and insulin resistance [27]. Moreover, acute stressors such as abrupt lifestyle modifications and disease‐related stress may compromise β‐cell regulatory mechanisms and functional capacity, thereby elevating the risk of hyperglycemia recurrence. To overcome these challenges, ongoing metabolic management, especially weight management, should be considered [28, 29, 30]. This is supported by our previous study showing that self‐management abilities and positive attitudes toward the disease are pivotal for maintaining remission after SIIT [31]. Notably, only modest weight reduction was observed across all treatment groups, similar to findings in REMIT‐sita and REMIT‐dapa studies [19, 20]. More aggressive weight management may be needed for durable diabetes remission. In addition, some studies have shown that prolonged medicine administration contributed to modification of the natural progression of type 2 diabetes mellitus. For instance, in the ADOPT study investigating metformin or rosiglitazone monotherapy in type 2 diabetes mellitus, the maximal effect on HbA1c and β‐cell function determined by HOMA2 was observed at 12 months [32]. In one of our recent studies, a simplified antihyperglycemic strategy following SIIT, particularly linagliptin plus metformin, resulted in more durable glycemic control and β‐cell function preservation after 48 weeks of follow‐up [33]. Thus, SIIT is a good start for reversing β‐cell dysfunction, but more prolonged sequential therapy may offer the best approach for maintaining long‐term glycemic control. The results of this study also provide information on subsequent medication selection. Patients receiving insulin sensitizers achieved superior glycemic control and β‐cell function improvements, consistent with our preliminary study [18] and previous reports, demonstrating that sequential treatment using metformin after SIIT surpassed intermittent IIT strategy in long‐term glycemic outcomes and β‐cell function [34, 35]. These findings support the use of insulin sensitizers in sequential treatment regimens for early type 2 diabetes mellitus, which warrant future study. Given the importance of weight control and insulin sensitivity in maintaining remission, agents including GLP‐1 RAs and SGLT‐2 inhibitors may serve as promising options within prolonged sequential therapy. Their integration could complement SIIT‐induced metabolic improvements and address mechanisms underlying relapse. Future studies are needed to determine optimal strategies for achieving durable glycemic control in early type 2 diabetes mellitus.

Strengths of this study included its randomized design and comprehensive longitudinal glycemic and metabolic assessments. Several limitations should be noted. First, this was an open‐label study without blinding of investigators and participants. Second, diet and exercise recommendations followed local guidelines rather than standardized protocols, enhancing real‐world applicability but limiting strict control. Third, medication adherence was assessed subjectively at each visit rather than using validated quantitative measures. Fourth, the sample size was calculated based on prior studies with larger anticipated treatment effects, which may have contributed to the negative results by underpowering the study. Finally, limited racial and ethnic diversity may restrict generalizability, and future studies should validate these findings in more diverse populations.

In conclusion, treatment with insulin sensitizers (metformin and pioglitazone) or sitagliptin for 3 months in combination with SIIT facilitated the implementation of tight glycemic control during SIIT and improved glycemic control and β‐cell function in the short term, but did not impact long‐term diabetes remission. Whether extended sequential treatments (especially those targeting insulin resistance and obesity) after SIIT can better preserve β‐cell function and long‐term glycemic control requires future investigation.

## Author Contributions

W.K., L.L., and P.Z. contributed equally as co‐first authors, with roles in data collection, statistical analysis, and manuscript drafting. L.Y., Q.Z., F.Z., and X.X. led participant recruitment and study implementation at their respective sites. Y.L., W.K., L.L., P.Z., L.Y., Q.Z., F.Z., X.X., J.L., L.X., X.W., H.L., X.C., and H.X. contributed to the execution of the trial. Y.L. conceived the study design, supervised the research implementation, led interpretation of the key findings, and critically reviewed the manuscript. M.S.P. provided expertise in data interpretation, and made substantial contributions to manuscript revision. Y.L. and M.S.P. contributed equally as co‐corresponding authors. All authors have seen and approved the final manuscript.

## Funding

This work was supported by the National Key R&D Program of China (2018YFC1314100), The Key‐Area Research and Development Program of Guangdong Province (2019B020230001), Sun Yat‐sen University Clinical Research 5010 Program, The National Natural Science Fund of China (81800716, 81870557), The Science and Technology Program of Guangzhou (202002020053, 2023A04J2190).

## Conflicts of Interest

M.S.P. has received research funding and honoraria from Vertex Pharmaceuticals, investigator‐initiated research funding from Dexcom and Samsung, and serves on the scientific advisory board of Anagram Therapeutics. All other authors report no conflicts of interest to be declared.

## Supporting information


**Table S1:** Daily basal insulin dosages during SIIT (IU/day, mean ± SD).
**Table S2:** Daily bolus insulin dosages during SIIT (IU/day, mean ± SD).
**Table S3:** Daily total insulin dosages during SIIT (IU/day, mean ± SD).
