# Supplementary material for: Effects of Short‐Term Intensive Insulin Therapy Combined With Oral Hypoglycemic Agents for Inducing Remission in Newly Diagnosed Type 2 Diabetes Mellitus: A Randomized Clinical Trial
Source: J Diabetes. 2026 Jan 11;18(1):e70187. doi: 10.1111/1753-0407.70187 (PMC12790882; doi:10.1111/1753-0407.70187)
Supplement: Supplementary file 1 — Table S1: Daily basal insulin dosages during SIIT (IU/day, mean ± SD). Table S2: Daily bolus insulin dosages during SIIT (IU/day, mean ± SD). Table S3: Daily total insulin dosages during SIIT (IU/day, mean ± SD). [file JDB-18-e70187-s001.docx]

**Supplementary Material**

1. **Daily insulin dosages during short-term intensive insulin therapy (SIIT)**

1.1 Table S1 Daily basal insulin dosages during SIIT (IU/d, mean±SD)

1.2 Table S2 Daily bolus insulin dosages during SIIT (IU/d, mean±SD)

|  | CSII+Met+Pio group | CSII+Sita group | CSII group | P |
| --- | --- | --- | --- | --- |
| D01 | 21.60(17.50, 26.08) | 21.60(16.80, 26.40) | 22.55(18.00, 28.80) | 0.328 |
| D02 | 19.80(16.80, 24.60) | 19.80(15.90, 25.90) | 21.60(15.51, 28.88) | 0.479 |
| D03 | 18.55(14.58, 22.45) | 19.50(14.70, 25.10) | 21.60(15.33, 28.80) | 0.258 |
| D04 | 16.80(13.28, 21.93) | 17.55(13.60, 24.00) | 19.75(14.48, 27.83) | 0.178 |
| D05 | 15.40(12.00, 21.05) | 17.20(12.90, 23.45) | 18.55(13.18, 26.40) | 0.166 |
| D06 | 14.40(10.65, 19.93) | 16.10(12.00, 22.30) | 16.65(13.08, 24.85) | 0.051 |
| D07 | 13.20(9.60, 18.88) | 15.00(9.90, 21.60) | 16.15(12.00, 23.48) | 0.042 |
| D08 | 12.00(8.43, 17.65) | 14.10(9.30, 21.30) | 15.73(11.24, 21.08) | 0.048 |
| D09 | 12.00(7.80, 17.03) | 13.60(8.00, 21.30) | 14.70(10.59, 19.20) | 0.035 |
| D10 | 11.00(7.01, 16.20) | 12.90(7.80, 21.30) | 13.95(10.03, 18.73) | 0.031 |
| D11 | 9.60(6.80, 15.00) | 12.60(7.20, 20.20) | 12.88(9.10, 16.98) | 0.016 |
| D12 | 9.15(6.14, 13.08) | 12.00(7.20, 19.00) | 12.40(8.20, 15.75) | 0.012 |
| D13 | 8.55(4.80, 12.00) | 12.00(6.50, 18.15) | 12.00(8.08, 15.15) | 0.006 |
| D14 | 8.40(4.80, 12.00) | 11.60(6.50, 18.15) | 12.00(7.95, 14.55) | 0.005 |

|  | CSII+Met+Pio group | CSII+Sita group | CSII group | P |
| --- | --- | --- | --- | --- |
| D01 | 24.25(20.63, 30.00) | 25.00(20.10, 30.50) | 29.50(23.00, 34.00) | 0.001 |
| D02 | 23.00(18.25, 28.50) | 24.00(19.50, 32.00) | 29.00(23.88, 35.13) | <0.001 |
| D03 | 21.50(18.00, 27.88) | 23.00(19.00, 30.00) | 28.00(23.00, 35.13) | <0.001 |
| D04 | 20.50(16.13, 26.00) | 22.00(18.50, 29.00) | 28.00(22.00, 34.13) | <0.001 |
| D05 | 20.00(15.00, 25.38) | 21.90(17.00, 29.00) | 28.00(21.00, 33.00) | <0.001 |
| D06 | 18.75(13.13, 24.00) | 20.50(16.00, 28.00) | 25.00(20.00, 32.00) | <0.001 |
| D07 | 17.00(12.50, 22.88) | 20.00(16.00, 27.00) | 25.00(18.88, 31.00) | <0.001 |
| D08 | 16.00(12.00, 21.63) | 20.00(15.50, 26.00) | 24.00(17.88, 29.25) | <0.001 |
| D09 | 15.00(11.25, 21.00) | 19.50(14.00, 25.50) | 22.50(17.00, 27.00) | <0.001 |
| D10 | 13.75(10.00, 19.75) | 18.00(13.00, 25.00) | 22.00(16.75, 26.13) | <0.001 |
| D11 | 13.25(9.00, 19.00) | 17.00(13.00, 24.00) | 20.00(14.38, 25.13) | <0.001 |
| D12 | 12.00(9.00, 17.00) | 16.00(12.00, 24.00) | 18.75(13.38, 24.25) | <0.001 |
| D13 | 12.00(8.13, 16.00) | 16.00(11.00, 23.00) | 17.00(12.00, 23.63) | <0.001 |
| D14 | 12.00(8.00, 16.00) | 16.00(10.50, 22.00) | 16.75(12.00, 24.00) | <0.001 |

1.3 Table S3 Daily total insulin dosages during SIIT (IU/d, mean±SD)

|  | CSII+Met+Pio group | CSII+Sita group | CSII group | P |
| --- | --- | --- | --- | --- |
| D01 | 44.75(39.63, 55.65) | 45.30(38.80, 56.00) | 50.25(43.48, 62.25) | 0.008 |
| D02 | 43.30(35.50, 53.93) | 37.60(44.90, 53.50) | 50.00(41.45, 63.94) | 0.005 |
| D03 | 40.20(33.40, 49.68) | 43.80(36.30, 53.00) | 48.75(41.20, 61.35) | 0.001 |
| D04 | 36.65(31.13, 48.88) | 41.40(34.20, 51.15) | 47.10(38.30, 59.73) | <0.001 |
| D05 | 34.73(29.48, 47.10) | 40.00(32.00, 50.00) | 46.05(36.43, 57.88) | <0.001 |
| D06 | 33.20(26.33, 44.63) | 38.00(30.20, 46.80) | 43.25(33.98, 55.60) | <0.001 |
| D07 | 30.55(22.88, 40.28) | 36.30(28.60, 45.00) | 41.95(32.25, 52.33) | <0.001 |
| D08 | 28.80(22.03, 37.83) | 34.80(26.90, 43.60) | 41.18(29.69, 48.51) | <0.001 |
| D09 | 26.30(20.83, 35.53) | 33.20(26.15, 43.60) | 38.15(28.69, 46.36) | <0.001 |
| D10 | 23.75(18.98, 34.35) | 31.00(24.60, 42.60) | 34.95(28.09, 44.09) | <0.001 |
| D11 | 22.33(17.18, 32.30) | 29.40(22.60, 41.50) | 32.90(26.38, 42.60) | <0.001 |
| D12 | 20.90(16.11, 30.35) | 27.80(21.30, 39.50) | 30.85(24.59, 24.59) | <0.001 |
| D13 | 19.90(15.00, 28.48) | 27.30(20.80, 38.40) | 29.30(24.59, 38.15) | <0.001 |
| D14 | 19.65(14.55, 28.10) | 26.20(20.80, 38.40) | 29.15(23.08, 38.15) | <0.001 |
